# Provider preferences for delivery of HIV care coordination services: results from a discrete choice experiment

**DOI:** 10.1002/jia2.25887

**Published:** 2022-03-24

**Authors:** Rebecca Zimba, Chunki Fong, Madellena Conte, Abigail Baim‐Lance, McKaylee Robertson, Jennifer Carmona, Gina Gambone, Denis Nash, Mary Irvine

**Affiliations:** ^1^ Institute for Implementation Science in Population Health (ISPH) City University of New York (CUNY) New York USA; ^2^ Department of Epidemiology and Biostatistics Graduate School of Public Health and Health Policy City University of New York (CUNY) New York USA; ^3^ Department of Epidemiology and Biostatistics Institute for Implementation Science in Population Health (ISPH) City University of New York (CUNY) New York USA; ^4^ Bureau of Hepatitis, HIV and Sexually Transmitted Infections New York City Department of Health and Mental Hygiene New York USA

**Keywords:** discrete choice experiment, HIV, antiretroviral therapy, adherence, care coordination, New York City

## Abstract

**Introduction:**

The PROMISE study was launched in 2018 to assess and document the implementation of changes to an existing HIV Care Coordination Programme (CCP) designed to address persistent disparities in care and treatment engagement among persons with HIV in New York City. We evaluated provider endorsement of features of the CCP to understand drivers of engagement with the programme.

**Methods:**

We used a discrete choice experiment to measure provider endorsement of four CCP attributes, including: (1) how CCP helps with medication adherence, (2) how CCP helps with primary care appointments, (3) how CCP helps with issues other than primary care and (4) where CCP visits take place (visit location). Each attribute had three to four levels. Our primary outcomes were relative importance and part‐worth utilities, measures of preference for the levels of the four CCP program attributes, estimated using a hierarchical‐Bayesian multinomial logit model. All non‐medical providers in the core CCP positions of patient navigator, care coordinator and programme director or other administrator from each of the 25 revised CCP‐implementing agencies were eligible to participate.

**Results:**

We received responses from 152 providers, 68% of whom identified as women, 49% identified as Latino/a, 34% identified as Black and 60% were 30–49 years old. Visit location (28.6%, 95% confidence interval [CI] 27.0–30.3%) had the highest relative importance, followed by how staff help with ART adherence (24.3%, 95% CI 22.4–26.1%), how staff help with issues other than primary care (24.2%, 95% CI 22.7–25.7%) and how staff help with primary care appointments (22.9%, 95% CI 21.7–24.1%). Within each of the above attributes, respectively, the levels with the highest part‐worth utilities were home visits 60 minutes from the program or agency (utility 19.9, 95% CI 10.7–29.0), directly observed therapy (utility 26.1, 95% CI 19.1–33.1), help with non‐HIV specialty medical care (utility 26.5, 95% CI 21.5–31.6) and reminding clients about and accompanying them to primary care appointments (utility 20.8, 95% CI 15.6–26.0).

**Conclusions:**

Ongoing CCP refinements should account for how best to support and evaluate the intensive CCP components endorsed by providers in this study.

## 1 INTRODUCTION

Antiretroviral therapy (ART) adherence improves clinical outcomes among persons with HIV (PWH) and reduces onward transmission [1, 2, 3, 4]. However, in the United States, the Centers for Disease Control and Prevention (CDC) estimates that only 65.5% of persons diagnosed with HIV in 2018 and alive at the end of 2019 had achieved viral suppression (≤200 copies/ml) by the end of 2019 [5]. In New York City (NYC), 77% of PWH were virally suppressed in 2018, though stratified viral suppression rates indicate persistent disparities across multiple subgroups, including age, sex, gender, race and transmission risk [6, 7, 8].

In 2009, the New York City Department of Health and Mental Hygiene (NYC Health Department) implemented a multi‐component HIV Care Coordination Programme (CCP) with the goal of improving engagement in care and treatment among the most vulnerable PWH in NYC, including those facing the additional challenges of mental health issues, food insecurity and unstable housing [9, 10]. The programme combined the following elements: outreach both for initial case‐finding and after missed appointments; case management; communication across disciplines within the care team; care decision making within case conferences; patient navigation; ART adherence support, including optional directly observed therapy (DOT); and structural health promotion [9]. The programme has since been included in the CDC's Compendium of Evidence‐Based Interventions and Best Practices for HIV Prevention [11, 12, 13].

The initial CCP was implemented at 28 Ryan White Part A‐funded agencies, reaching over 7000 clients in less than 4 years. The CCP demonstrated modest benefits for viral load suppression among newly diagnosed PWH and previously diagnosed but consistently unsuppressed PWH [10, 14, 15]. Following an outline developed by the NYC Health Department and the local Ryan White Part A Community Planning Council, refinements to the CCP were implemented in 2018 to enhance intervention delivery, engagement and impact, and to reduce implementation barriers. Modifications included adding a client self‐management assessment, a video chat visit option and the immediate initiation of ART following enrolment or diagnosis; changing guidance to promote the identification and recruitment of PWH with clinical need for care coordination; changing the payment structure from per‐client‐per‐day to fee‐for‐service; and increasing the programme's flexibility to allow for more differentiated care based on clients’ needs [16]. The program will likely continue to evolve.

The PROMISE study (Program Refinements to Optimize Model Impact and Scalability Based on Evidence) was launched in 2018 to assess and document the implementation of changes to the CCP; additional details can be found in the study's protocol paper [16]. Recognizing that successful programme implementation depends upon both client and provider engagement, we conducted two discrete choice experiments (DCEs) to understand client and provider preferences for specific programme features. Originating in econometrics [17], DCEs increasingly are being used in public health and healthcare to better understand drivers of uptake and engagement with evidence‐based interventions [18]. DCEs have been used to study HIV prevention, counselling, testing, care and treatment [19], for example to understand factors that may be important to retention among patients lost to care in Zambia [20], and to characterize preference for pre‐exposure prophylaxis programmes among men who have sex with men [21]. Here, we present the findings from our provider DCE.

## 2 METHODS

### 2.1 Population and sampling

Ryan White Part A funding in NYC is used to fund services other than medical care, therefore, our target population was enumerated from a census of all non‐medical providers in the core CCP positions of patient navigators/health educators, who work most closely with the clients and provide critical feedback to the care team to inform care planning; care coordinators/case managers, who lead comprehensive assessments, facilitate care team activities, such as case conferences, and supervise one or more patient navigators; and programme directors or other administrators at any of the 25 agencies implementing the revised CCP. All 227 staff in those core programme roles were eligible to participate. Ten agencies were community health centres, six were private hospitals, three were public hospitals and six were community‐based organizations. All were co‐located with or had formal partnerships with clinical facilities, with caseloads ranging from approximately 35 to 250. Five agencies were located in Brooklyn, nine were located in Manhattan, seven were located in the Bronx, three were located in Queens and one was located in Staten Island. The study protocols and materials were reviewed and approved by the NYC Health Department institutional review board. All participants provided informed consent electronically.

### 2.2 Developing the attributes and levels

We wanted to include aspects of the CCP that could be explored in both the current provider DCE and a subsequent client DCE in order to facilitate future concordance analyses. In accordance with best practices for designing DCEs [22, 23], we began developing a list of programme features to investigate in the DCE through two client focus groups (seven participants total) and one provider focus group (five participants). See Table S1 for participant details.

**Figure 1:**
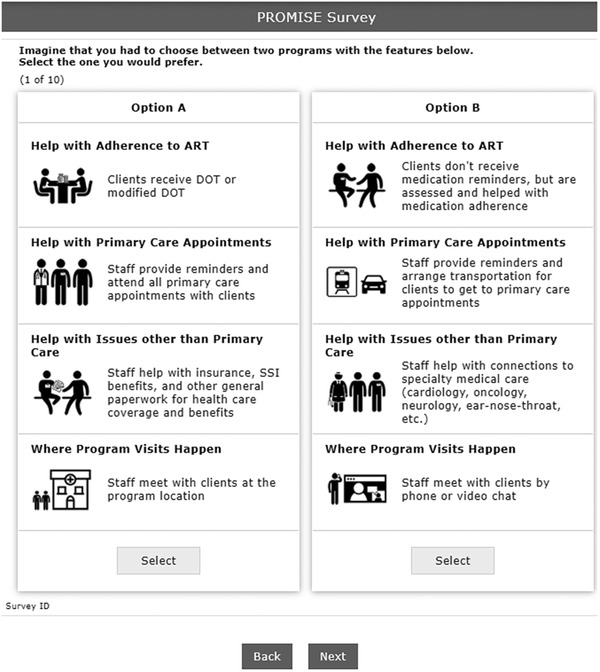
Example of discrete choice experiment (DCE) task presented to providers (desktop or laptop browser orientation). Abbreviations: ART, antiretroviral therapy; DOT, directly observed therapy; SSI, Supplemental Security Income, a federal programme that provides monthly payments to people with income below certain financial limits

We also considered which of the key elements of the programme might be amenable to future changes, whether in focus, intensity or mode of delivery. The possible features and versions of those features, called attributes and attribute levels in the parlance of DCEs, were originally drafted by reviewing focus group feedback and through discussion within the study team, and refined based on feedback from PROMISE Study Advisory Board members at a meeting in June 2019. Our final design included four attributes with three to four levels each, which varied by focus, intensity and/or mode: (1) help with adherence to ART; (2) help with primary care appointments; (3) help with issues other than primary care; and (4) where programme visits happen (visit location). It should be noted that though presented as mutually exclusive options within the DCE, all of the level options are supported by the CCP. Black‐and‐white graphics were included for each attribute level to facilitate quick comprehension and comparison of the attribute levels across choice concepts. See Table 1 and Figure 1.

**Table 1:** Attributes and levels of a discrete choice experiment investigating provider preferences for HIV care coordination services in New York City

Attribute	Attribute‐level description	Helper image
Help with adherence to ART	Clients receive DOT or modified DOT	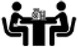
	Clients receive medication reminders by phone call or text	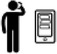
	Clients do not receive medication reminders, but are assessed and helped with medication adherence	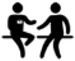
Help with primary care appointments	Staff provide reminders and attend all primary care appointments with clients	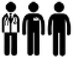
	Staff provide reminders and arrange transportation for clients to get to primary care appointments	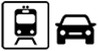
	Staff only provide reminders for primary care appointments	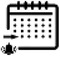
Help with issues other than primary care	Staff help with insurance, SSI benefits and other general paperwork for healthcare coverage and benefits	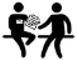
	Staff help with securing housing and food	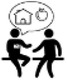
	Staff help with mental health and wellbeing issues (such as stress, substance use, diet or personal relationships)	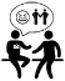
	Staff help with connections to specialty medical care (cardiology, oncology, neurology, ear‐nose‐throat, etc.)	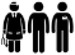
Where programme visits happen	Staff meet with clients at the programme location	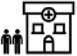
	Staff meet with clients by phone or video chat	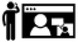
	Staff make home visits, 30 minutes from the programme location	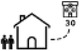
	Staff make home visits, 60 minutes from the programme location	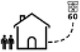

Abbreviations: ART, antiretroviral therapy; DOT, directly observed therapy; SSI, Supplemental Security Income, a federal programme that provides monthly payments to people with income below certain financial limits.

### 2.3 DCE design

The survey was designed and implemented using Lighthouse Studio Version 9.8.1 (Sawtooth Software, Provo, UT, USA) and deployed via Sawtooth's online survey hosting platform. The final design included 10 comparison tasks, with two alternatives per task; to improve design efficiency and the precision of our main effects part‐worth utility estimates, we chose not to include a “None” option. We used Sawtooth's Balanced Overlap method [24, 25] to generate random tasks in which each level appeared approximately the same number of times as the other levels within each attribute (level balance), some level overlap within an attribute was permitted across alternatives in the same task and levels within one attribute were included independently of levels within other attributes (orthogonality). Our design's relative D‐efficiency was 88% compared to the D‐efficiency of Sawtooth's Completely Enumerated design, which is statistically more efficient but is less able to identify possible interaction effects due to minimal overlap between alternatives within a choice task [24]. The survey was deployed in English.

Introductory text was included to describe the attributes being investigated in the survey and explained that “Your responses will tell us what programme features providers value most and what features they might like to change.” In each choice exercise, we asked providers to “Imagine that you had to choose between two programmes with the features below. Select the one that you would prefer.” After the choice exercises, we asked respondents about their age, race/ethnicity and gender identity, and the length of time they had been providing CCP services.

### 2.4 Sample size

The minimum sample size for estimating main effects in a DCE can be calculated as $n\geq \frac{500c}{ta}$, where n is the number of respondents, c is the maximum number of levels among all of the attributes, t is the number of choice tasks and a is the number of alternatives per task [26, 27]. This formula assumes each main‐effect level appears at least 500 times within the survey design. The minimum sample size for our study given a maximum of four levels among our attributes, 10 choice tasks and two alternatives per task is $\frac{500(4)}{\left(10)(2\right)}=\;100$, therefore, our target sample size of 150 responses was sufficient to estimate main effects.

### 2.5 Data collection

In January 2020, we emailed the survey link and individual survey IDs to the 227 eligible providers in core CCP roles from the 25 agencies implementing the revised CCP, with a target sample of 150 completed responses. The DCE could be completed in any modern browser on a desktop or laptop computer, tablet or phone; on mobile devices, participants swiped horizontally to compare the choice concepts. Participants were compensated with $25 gift cards upon survey completion. The survey was closed in early March 2020, before the first wave of the COVID‐19 pandemic in NYC, with 152 respondents. Into the final survey data set, we merged additional staff and agency descriptive data, such as staff role, agency location and CCP budget, gathered from programme liaisons and existing NYC Health Department contract records.

### 2.6 Analysis

We used Sawtooth Software's Lighthouse Studio 9.8.1 to analyse the survey. We estimated part‐worth utilities for each attribute level using the hierarchical‐Bayesian multinomial logit (HB) method and assuming the Random Utility Model, which posits that people choose the option that has the highest total utility for them [28]. The HB method analyses the data at the individual level and the aggregate level, which yields more stable respondent‐level estimates and also allows for more heterogeneity across respondents than other methods, such as traditional multinomial logit regression [29, 30]. See Text S1 for methods used to assess model fit and respondent quality.

### 2.7 Part‐worth utilities and relative importance

We interpreted part‐worth utilities as preferences for or endorsements of CCP features. We estimated zero‐centred utilities at the individual level using effects coding, in which utilities are rescaled so that the reference level is the negative sum of the utilities of the other levels within each attribute [17, 28]. We calculated the mean of the utilities across individuals and 95% confidence intervals as the mean ± 1.96 × standard error. Attribute relative importance quantifies the degree to which an attribute influences choices relative to the other attributes. We calculated relative importance scores for each attribute and each respondent as the range in utilities for levels within an attribute over the sum of the ranges in utilities for levels in all attributes. We averaged this respondent‐level measure to get an aggregate‐level measure of attribute relative importance, scaled from 0 to 1, with a 95% confidence interval calculated in the same manner as above. Mean utilities, importances and 95% confidence intervals were calculated by Sawtooth. Distribution of participant characteristics was tabulated in SAS 9.4 (Cary, NC, USA).

## 3 RESULTS

### 3.1 Respondent demographics and agency characteristics

Characteristics of the 152 respondents are described in Table 2. Though we had no *a priori* hypotheses about the influence of gender on providers’ preferences, we report characteristics disaggregated by gender identity to provide a richer description of the respondents. At least one provider responded from each of the 25 CCP agencies (median 6 respondents and IQR 4–7 respondents). Providers who responded to the survey were primarily Black (34%) or Latino/a (49%), identified as women (68%) and were between 30 and 49 years old (60%). Most respondents were patient navigators (65%) and had worked in care coordination for over 2 years (58%). The agencies at which most respondents worked were based in Manhattan (34%), Bronx (28%) or Brooklyn (24%), were clinic based (84%) and had experience with the initial and revised care coordination model (76%). One hundred and forty providers completed the DCE on computers, 11 on phones and one on a tablet. See Text S1 for results regarding model fit and respondent quality.

**Table 2:** Demographic and agency characteristics of participating providers by gender identity (*N* = 152)

	All		Gender identity					
			Woman		Man		Other^a^	
	*N*	%	*N*	%	*N*	%	*N*	%
Overall^b^	152	100	104	68	43	29	5	3
Age group								
20–29	20	13	18	17	1	2	1	20
30–39	59	39	36	35	21	49	2	40
40–49	32	21	24	23	7	16	1	20
50–59	29	19	18	17	10	23	1	20
60 or older	12	8	8	8	4	9	0	0
Race/ethnicity								
Asian	6	4	5	5	1	2	0	0
Black	51	34	39	38	10	23	2	40
Latino/Latina	74	49	45	43	28	65	1	20
White	12	8	8	8	2	5	2	40
Multi‐racial	2	1	1	1	1	2	0	0
Other	4	3	3	3	1	2	0	0
Missing	3	2	3	3	0	0	0	0
Role								
Navigator‐type staff	99	65	64	62	30	70	5	100
Administrative staff	20	13	13	13	7	16	0	0
Care coordinator‐type staff	33	22	27	26	6	14	0	0
When respondent began providing care coordination services								
Less than 6 months ago	9	6	6	6	2	5	1	20
6 months to 1 year ago	21	14	14	13	7	16	0	0
1–2 years ago	34	22	21	20	12	28	1	20
More than 2 years ago	88	58	63	61	22	51	3	60
Agency borough								
Bronx	42	28	34	33	8	19	0	0
Brooklyn	37	24	26	25	11	26	0	0
Manhattan	52	34	28	27	19	44	5	100
Queens	14	9	12	12	2	5	0	0
Staten Island	7	5	4	4	3	7	0	0
Agency location								
Clinic based	128	84	86	83	37	86	5	100
Non‐clinic	24	16	18	17	6	14	0	0
Agency care coordination program experience								
Experienced	116	76	83	80	28	65	5	100
New	36	24	21	20	15	35	0	0
Care coordination budget in thousands, calendar year 2019								
Median (IQR)	$758 ($610–$876)		$758 ($610–$892)		$695 ($617–$800)		$526 ($474–$892)	
Mean (SD)	$790 ($253)		$808 ($265)		$760 ($216)		$684 ($298)	

^a^ Other includes transwoman, transman, gender non‐conforming or non‐binary and other.

^b^ Row percents. All others column percents.

Abbreviations: IQR, interquartile range; SD, standard deviation.

### 3.2 Relative importance

Relative importance estimates for each attribute are shown in Table 3. Visit location had the highest relative importance (28.6%, 95% CI 27.0–30.3%), followed by how staff help with ART adherence (24.3%, 95% CI 22.4–26.1%), how staff help with issues other than primary care (24.2%, 95% CI 22.7–25.7%) and lastly how staff help with primary care appointments (22.9%, 95% CI 21.7–24.1%). Only the confidence interval for visit location did not overlap with the confidence intervals of the other three attributes, which all overlapped with each other. Relative importance estimates for each attribute stratified by gender identity are shown in Table S2.

**Table 3:** Average relative attribute importance from a discrete choice experience among providers in New York City assessing preference for HIV care coordination programme features

Attribute	Average relative importance	Standard deviation	Lower 95% CI	Upper 95% CI
How staff help with ART adherence	24.3%	11.7%	22.4%	26.1%
How staff help with primary care appointments	22.9%	7.8%	21.7%	24.1%
How staff help with issues other than primary care	24.2%	9.6%	22.7%	25.7%
Visit location	28.6%	10.3%	27.0%	30.3%

Abbreviation: ART, antiretroviral therapy.

### 3.3 Part‐worth utilities

**Figure 2:**
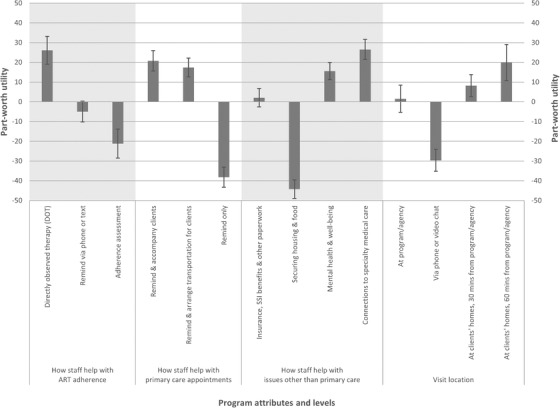
Part‐worth utilities^†^ from a discrete choice experience among providers in New York City assessing preference for HIV care coordination programme features. ^†^Part‐worth utilities were estimated using effects coding and are zero‐centred. Abbreviations: ART, antiretroviral therapy; SSI, Supplemental Security Income, a federal programme that provides monthly payments to people with income below certain financial limits.

Part‐worth utilities of levels within each attribute are shown in Table 4 and Figure 2. The magnitude and direction of part‐worth utilities indicate the strength of preference or endorsement for levels within an attribute; negative utilities do not connote aversion or dislike, merely lower preference relative to the other levels in the attribute. Providers preferred programmes that included DOT as a strategy to help with ART adherence (part‐worth utility 26.1, 95% CI 19.1–33.1), compared to reminding clients to take ART via phone or text (–5.0, 95% CI –10.2 to 0.3) and only assessing and helping with ART adherence based on responses to assessments (–21.1, 95% CI –28.5 to –13.8). Providers preferred programmes that offered reminders about and accompaniment to primary care appointments (20.8, 95% CI 15.6–26.0) and those that reminded about and arranged transportation for primary care appointments (17.4, 95% CI 12.7–22.2), over those that only offered reminders about primary care appointments (–38.2, 95% CI –43.3 to –33.0). Providers preferred programmes that focused on helping clients with connections to specialty medical care for health conditions other than HIV (26.5, 95% CI 21.5–31.6) or with mental health and wellbeing (15.6, 95% CI 11.2–20.0), compared with programmes that focused on helping with insurance, Supplemental Security Income and other benefits paperwork (2.1, 95% CI –2.6 to 6.8) or helping with securing housing and food (–44.3, 95% CI –48.9 to –39.6). Lastly, providers selected programmes capable of providing home visits at locations up to 60 minutes away from the programme or agency location (19.9, 95% CI 10.7–29.0), and home visits up to 30 minutes away from the programme or agency location (8.2, 95% CI 2.6–13.8) more than programmes in which staff only met clients at the programme or agency (1.6, 95% CI –5.3 to 8.5) or met clients only via phone or video chat (–29.6, 95% CI –35.8 to –24.1). Part‐worth utilities of levels within each attribute stratified by gender identity are shown in Table S3.

**Table 4:** Part‐worth utilities^a^ from a discrete choice experience among providers in New York City assessing preference for HIV care coordination programme features

Attribute	Level	Utility	Standard deviation	Lower 95% CI	Upper 95% CI
How staff help with ART adherence	Directly observed therapy	26.1	44.1	19.1	33.1
	Reminder via phone or text	–5.0	33.1	–10.2	0.3
	Adherence assessment	–21.2	46.1	–28.5	–13.8
How staff help with primary care appointments	Remind and accompany clients	20.8	32.5	15.6	26.0
	Remind and arrange transportation for clients	17.4	29.9	12.7	22.2
	Remind only	–38.2	32.4	–43.3	–33.0
How staff help with issues other than primary care	Insurance, SSI benefits and other paperwork	2.1	29.3	–2.6	6.8
	Securing housing and food	–44.3	29.3	–48.9	–39.6
	Mental health and wellbeing	15.6	27.4	11.3	20.0
	Connections to specialty medical care	26.5	32.1	21.5	31.6
Visit location	At programme/agency	1.6	43.4	–5.3	8.5
	Via phone or video chat	–29.6	34.8	–35.8	–24.1
	At clients' homes, 30 minutes from programme/agency	8.2	35.3	2.6	13.8
	At clients' homes, 60 minutes from programme/agency	19.9	57.5	10.7	29.0

^a^ Part‐worth utilities were estimated using effects coding and are zero‐centred.

Abbreviations: ART, antiretroviral therapy; SSI, Supplemental Security Income, a federal programme that provides monthly payments to people with income below certain financial limits.

## 4 DISCUSSION

Our research adds to a growing body of knowledge around patient‐ and provider‐stated preferences for HIV prevention, counselling, testing, care and treatment [19, 20, 21, 31, 32, 33, 34, 35]. Many studies focus on the clinical aspects of HIV care and treatment (viral load testing location and frequency, ART refill frequency, etc.), though some assess preferences for features of care coordination. One study used best‐worst scaling, a kind of conjoint survey and analysis method, to examine provider preferences for healthcare services for HIV‐hepatitis C‐coinfected patients in a safety‐net hospital in San Francisco [35]. In this study, providers endorsed the provision of support services, such as medication and appointment reminders; these are similar to our findings, though providers in our study preferred DOT over medication reminders alone, and accompanying clients or arranging transportation for clients over appointment reminders alone. Another study examined provider preferences for differentiated service delivery and ART maintenance services in Thailand, finding that providers endorsed longer ART refill visit spacing and the decentralization of ART maintenance services, as well as psychosocial support [32]. These findings align with our own; though many of these studies varied the frequency of services, such as viral load testing and ART refills, none explicitly varied the intensity of the service, and none included items specifically related to DOT.

Among providers in New York City who took this survey about hypothetical variations on the HIV Care Coordination programme, all four categories of programme features had nearly equivalent relative importances, suggesting that providers’ choices were influenced similarly by all four attributes. However, the part‐worth utilities indicate that more intensive versions of services, such as DOT or accompanying clients to primary care appointments, were preferred over the less intensive alternatives, such as medication or appointment reminders alone.

Background viral load suppression in NYC is high compared to the country as a whole, with 77% of PWH achieving viral load suppression in NYC in 2018 [5, 8]. However, the population for which the CCP is intended comprises PWH who have either documented risks for poor HIV outcomes, a clear history of poor HIV outcomes or both. Though modelling did not find scaling‐up the intervention to be cost‐effective [36], our findings suggest that providers “on the ground” recognize and are willing to engage with the high level of service intensity required to reach these clients and improve ART adherence.

Regarding the preference for home visits, providers may have been affirming both the CCP model's value in filling service gaps in usual agency‐based care and an aspect of the programme that makes it effective for clients, who may live up to 60 minutes from the agency location if public transportation options are limited. This may be in recognition of the value of working with clients at their homes or in the field, regardless of the distance from the clinic.

Like home‐based visits, DOT is time‐intensive and costly, and is uncommon in other adherence‐support programmes. In the original CCP, clients were assigned to enrolment tracks, which determined the frequency and type of services they received. After the redesign in 2018, providers had more flexibility to adjust the frequency, type and intensity of services based on periodic assessments of individual client needs. The redesign also included a virtual DOT option. This expanded access to DOT for clients and reduced barriers to providing DOT for agencies. During the 2019 grant year (March 2019–February 2020), 14.2% of enrolled CCP clients received at least one DOT service, defined as the observation of a single dose, up from 7.7% during the 2017 grant year (March 2017–February 2018) [37].

Coordinating specialty medical care for non‐HIV health conditions or engaging clients in mental health and wellbeing services are activities that providers are well‐positioned to undertake in the CCP, relative to other case management programmes. However, our findings do not imply that providers devalue conventional case management activities. Because of how our DCE was designed, the levels within the “Help with Issues other than Primary Care” attribute were mutually exclusive; in real life, a care coordination programme could include support for housing and food *and* support for mental health and wellbeing. In fact, in recognition of the importance of supporting the whole client, the revised CCP includes reimbursable services related to helping with benefits and linking clients to housing and food services along with reimbursable services related to mental health and wellbeing. In this way, the CCP promotes the coordination of services across the social services and medical care systems to support the whole client. Similarly, this holistic approach to client care is reflected in the relative preferences for ways to provide more active assistance with primary care appointments.

### 4.1 Limitations

Our study has limitations, which should be acknowledged. Our sample size was sufficient for estimating main effects; however, the standard deviations of the part‐worth utility estimates indicate heterogeneity, which could be a consequence of factors, such as characteristics of providers or sites, or unmeasured variables pertaining to the client populations served at the agencies. Here, we reported the aggregate findings with the intention of examining heterogeneity in a separate latent class analysis; given the sample size, the latter analysis will be largely exploratory.

While we met our goal of 150 responses, this represents only two‐thirds of all eligible providers. Individual staff demographic data are not routinely collected by the NYC Health Department, which prevented us from comparing the frequencies of characteristics between respondents and non‐respondents. Our survey's respondents may not represent the demographics or agency characteristics, or the full spectrum of preferences, of all CCP providers in New York City Ryan White Part A agencies, which may limit the generalizability of our findings. However, all 25 care coordination‐delivering agencies and all core care coordination staff roles were represented among the study participants.

Our study was constrained by the DCE design, which must balance obtaining valuable and actionable data with limiting respondent cognitive fatigue. While including more or other attributes and levels would have yielded different findings, the attributes and levels in our study design capture the CCP features considered important by providers and clients of care coordination as ascertained through our focus groups.

Finally, our ability to interpret our findings is somewhat limited by the non‐specific language which framed the comparison tasks and by the reliance on survey self‐administration. Some providers may have made choices based on what they thought would make the programme better for clients, while others may have been thinking more about what makes the programme work for themselves or their agencies. Since either or both of these perspectives (benefit to clients or benefit to staff and agencies) could motivate providers to deliver the services they preferred in the DCE, and since the most preferred services were uniformly the more intensive options presented, we believe we may interpret our findings as indicating endorsement of and positive engagement with the unique and intensive features of the CCP.

## 5 CONCLUSIONS

The CCP fills gaps in an often‐fragmented service system through comprehensive coordination of healthcare and psychosocial support services, and the revised CCP allows for more flexible differentiated care based on client needs. We found consistent endorsement of the intensive client‐focused features that are rare in case management‐type programmes. We believe our findings show that providers particularly value the availability of an array of flexible care coordination features that have the potential to make the greatest difference for the most vulnerable clients. In response to these findings, revisions to consider making to the CCP could aim to enhance the sustainability of the delivery of the CCP's labour‐intensive features. Future work by this team will explore preference heterogeneity among providers using a latent class analysis, preferences among clients, the adoption of specific components of the CCP based on site reporting and programme utilization by client characteristics.

## COMPETING INTERESTS

There are no competing interests.

## AUTHORS' CONTRIBUTIONS

DN and MI conceptualized the study. ABL conducted formative work. ABL, DN, MI and RZ collaborated on the design of the data collection tool. RZ and CF performed statistical analyses. RZ, CF and MC wrote the first draft of the paper. RZ, CF, MC, ABL, MR, JC, GG, DN and MI contributed to interpreting the data and to the writing and revising of the manuscript.

## FUNDING

Research reported in this publication was supported by the National Institute of Mental Health of the National Institutes of Health under Award Number R01MH117793.

## DISCLAIMER

The content is solely the responsibility of the authors and does not necessarily represent the official views of the National Institutes of Health.

## Supporting information


**Table S1**: Characteristics of focus group attendees

**Table S2**: Average relative attribute importance by gender identity from a discrete choice experience among providers in New York City assessing preference for HIV care coordination programme features

**Table S3**: Part‐worth utilities by gender identity from a discrete choice experience among providers in New York City assessing preference for HIV care coordination programme features

**Text S1**: Model fit and respondent data quality

## Data Availability

Data will be made available upon request.
