# Supplementary material for: Provider preferences for delivery of HIV care coordination services: results from a discrete choice experiment
Source: J Int AIDS Soc. 2022 Mar 24;25(3):e25887. doi: 10.1002/jia2.25887 (PMC8944220; doi:10.1002/jia2.25887)
Supplement: Supplementary file 1 — Table S1: Characteristics of focus group attendees [file JIA2-25-e25887-s001.pdf]

Supplementary Table 1. Characteristics of focus group attendees

	Clients[†] (N=7)	Providers[‡] (N=5)
**Borough of residence/agency**		
Bronx	3	2
Brooklyn	0	1
Manhattan	0	2
Queens	2	0
Staten Island	0	0
Unknown	2	0
**Age**		
30-39	0	2
40-49	0	1
50-59	2	1
60 or older	3	1
Unknown	2	0
**Years in CCP**		
1-2 years	1	1
>2 years	4	4
Unknown	2	0
**Gender**		
Man	1	1
Woman	4	4
Unknown	2	0
**Race/ethnicity**		
Black	3	3
Latino/Latina	2	1
White	0	1
Unknown	2	0

[†]2 client groups on March 19 and 20, 2019 (n=7; 5, 2)
[‡]1 provider group on March 21, 2019 (n=5)
