# Supplementary material for: Provider preferences for delivery of HIV care coordination services: results from a discrete choice experiment
Source: J Int AIDS Soc. 2022 Mar 24;25(3):e25887. doi: 10.1002/jia2.25887 (PMC8944220; doi:10.1002/jia2.25887)
Supplement: Supplementary file 2 — Table S2: Average relative attribute importance by gender identity from a discrete choice experience among providers in New York City assessing preference for HIV care coordination programme features [file JIA2-25-e25887-s003.pdf]

Supplementary Table 2. Average relative attribute importance by gender identity from a discrete choice experience among providers in New York City assessing preference for HIV care coordination programme features

	All (N=152)				Gender Identity: Woman (N=104)				Gender Identity: Man (N=43)				Gender Identity: Other (N=5)			
**Attribute**	**Average Relative Importance**	**Std Dev**[†]	**Lower 95% CI**[‡]	**Upper 95% CI**	**Average Relative Importance**	**Std Dev**	**Lower 95% CI**[‡]	**Upper 95% CI**	**Average Relative Importance**	**Std Dev**	**Lower 95% CI**[‡]	**Upper 95% CI**	**Average Relative Importance**	**Std Dev**	**Lower 95% CI**[‡]	**Upper 95% CI**
How staff help with ART[§] adherence	24.3%	11.7%	22.4%	26.1%	24.8%	11.9%	22.5%	27.2%	23.2%	10.9%	19.9%	26.5%	22.0%	14.9%	3.5%	40.5%
How staff help with primary care appointments	22.9%	7.8%	21.7%	24.1%	22.8%	7.7%	21.3%	24.3%	22.7%	8.0%	20.3%	25.2%	26.2%	8.1%	16.1%	36.3%
How staff help with issues other than primary care	24.2%	9.6%	22.7%	25.7%	23.4%	8.9%	21.7%	25.1%	25.7%	10.8%	22.4%	29.0%	27.3%	11.7%	12.7%	41.9%
Visit location	28.6%	10.3%	27.0%	30.3%	29.0%	10.0%	27.0%	30.9%	28.4%	11.3%	24.9%	31.9%	24.5%	6.1%	16.9%	32.0%

[†]Std Dev - Standard Deviation

[‡]CI – Confidence interval

[§]ART – Antiretroviral therapy
