# Supplementary material for: Provider preferences for delivery of HIV care coordination services: results from a discrete choice experiment
Source: J Int AIDS Soc. 2022 Mar 24;25(3):e25887. doi: 10.1002/jia2.25887 (PMC8944220; doi:10.1002/jia2.25887)
Supplement: Supplementary file 3 — Table S3: Part‐worth utilities by gender identity from a discrete choice experience among providers in New York City assessing preference for HIV care coordination programme features [file JIA2-25-e25887-s002.pdf]

Supplementary Table 3. Part-worth utilities† by gender identity from a discrete choice experience among providers in New York City assessing preference for HIV care coordination programme features

		All (N=152)				Gender Identity: Woman (N=104)				Gender Identity: Man (N=43)				Gender Identity: Other (N=5)			
**Attribute**	**Level**	**Utility**	**Standard Deviation**	**Lower 95% CI§**	**Upper 95% CI**	**Utility**	**Standard Deviation**	**Lower 95% CI§**	**Upper 95% CI**	**Utility**	**Standard Deviation**	**Lower 95% CI§**	**Upper 95% CI**	**Utility**	**Standard Deviation**	**Lower 95% CI§**	**Upper 95% CI**
**How staff help with ART‡ adherence**	Directly observed therapy	26.1	44.1	19	33.2	26.2	45	17.5	35	28.9	39.6	16.7	41.1	-0.7	61.7	-77.2	75.9
	Reminder via phone or text	-4.9	33.1	-10.3	0.4	-5.1	34.1	-11.7	1.5	-3.2	31.9	-13	6.6	-17	24	-46.8	12.8
	Adherence assessment	-21.1	46.1	-28.5	-13.8	-21.1	47.5	-30.4	-11.9	-25.7	41.2	-38.4	-13	17.7	46.5	-40.1	75.4
**How staff help with Primary care appointments**	Remind & accompany clients	20.8	32.5	15.6	26	18.6	33.6	12.1	25.2	23.4	29.5	14.3	32.4	43	29.9	5.9	80.1
	Remind & arrange transportation for clients	17.4	29.9	12.6	22.2	16.7	31.2	10.6	22.8	21.7	26.3	13.6	29.8	-5.1	24.3	-35.2	25.1
	Remind only	-38.2	32.4	-43.4	-33	-35.4	34.3	-42	-28.7	-45.1	24.7	-52.7	-37.5	-37.9	47.5	-96.9	21.1
**How staff help with issues other than primary care**	Insurance, SSI¶ benefits & other paperwork	2.1	29.3	-2.6	6.8	1.2	27.7	-4.2	6.6	4.9	32.6	-5.1	15	-4.5	36.1	-49.3	40.3
	Securing housing & food	-44.3	29.3	-49	-39.6	-43.3	29	-48.9	-37.7	-45.6	31.6	-55.3	-35.8	-52.9	11.7	-67.5	-38.4
	Mental health & well-being	15.6	27.4	11.2	20	16	27.4	10.6	21.3	15.2	28.8	6.3	24.1	11.4	15.2	-7.5	30.3
	Connections to specialty medical care	26.5	32.1	21.4	31.7	26.1	29.1	20.4	31.7	25.4	37	14	36.8	46	45.9	-10.9	103
**Visit location**	At programme/agency	1.6	43.4	-5.4	8.5	4.7	43.7	-3.8	13.2	-6.1	42	-19	6.9	1.2	49.1	-59.8	62.1
	Via phone or video chat	-29.6	34.8	-35.2	-24.1	-28.5	37.4	-35.8	-21.2	-32.1	29.6	-41.2	-23	-32.2	20.3	-57.5	-7
	At clients' homes, 30 minutes from programme/agency	8.2	35.3	2.5	13.9	9	34.9	2.2	15.8	4	36.4	-7.2	15.2	27.2	33.4	-14.3	68.6
	At clients' homes, 60 minutes from programme/agency	19.9	57.5	10.7	29.1	14.7	59.5	3.2	26.3	34.2	53.5	17.7	50.6	3.9	25.6	-27.9	35.7

†Part-worth utilities were estimated using effects coding and are zero-centred

‡ART – Antiretroviral therapy

§CI – Confidence interval

¶SSI – Supplemental Security Income, a federal programme that provides monthly payments to people with income below certain financial limits
