# Supplementary material for: Provider preferences for delivery of HIV care coordination services: results from a discrete choice experiment
Source: J Int AIDS Soc. 2022 Mar 24;25(3):e25887. doi: 10.1002/jia2.25887 (PMC8944220; doi:10.1002/jia2.25887)
Supplement: Supplementary file 4 — Text S1: Model fit and respondent data quality [file JIA2-25-e25887-s004.pdf]

Model fit and respondent data quality

Methods

We measured overall model fit using percent certainty, analogous to McFadden's pseudo-R^2, where values from 0.2 to 0.4 indicate a good model fit [31]. We also used the model's overall root likelihood (RLH) to evaluate model fit. RLH is the geometric mean of the likelihood of each alternative within a choice task being selected, and ranges from $1/n$ (worst fit) to 1 (best fit), where n is the number of alternatives per task [32,33]. In this study, an RLH of 0.5 would indicate no model fit. We also assessed straightlining and response time for the DCE choice tasks, two potential indicators of respondent quality in DCEs [34].

Results

Though we did not have any *a priori* hypotheses about interactions between attribute levels, all possible interactions were explored, and none were found to be statistically significant. Overall fit for our model measured using McFadden's pseudo R^2 was 0.47, and RLH for our model was 0.69, both indicating better model fit over the null model. We conducted additional analyses excluding respondents identified through straightlining and fast response time. Median time to completion of the choice tasks was 395 seconds, or about 40 seconds per task (IQR 25 seconds-64 seconds per task). We categorized respondents as having response times less than 30 seconds per task (51 respondents), less than 20 seconds per task (21 respondents), and less than 10 seconds per task (4 respondents). Only 5 respondents exhibited straightlining choice behaviour. We found that dropping potentially poor-quality respondents from these additional analyses made no qualitative changes to part-worth utility or relative attribute importance estimates or our interpretation of the results.
